# The association between domestic animal presence and ownership and household drinking water contamination among peri-urban communities of Kisumu, Kenya

**DOI:** 10.1371/journal.pone.0197587

**Published:** 2018-06-06

**Authors:** Amber N. Barnes, John D. Anderson, Jane Mumma, Zahid Hayat Mahmud, Oliver Cumming

**Affiliations:** 1 Duke Global Health Institute, Duke University, Durham, North Carolina, United States of America; 2 Department of Environmental and Global Health, University of Florida, Gainesville, Florida, United States of America; 3 School of Natural Resources and the Environment, Emerging Pathogens Institute, University of Florida, Gainesville, Florida, United States of America; 4 Great Lakes University Kisumu, Kisumu, Kenya; 5 Environmental Microbiology Laboratory, International Centre for Diarrhoeal Disease Research, Bangladesh, Dhaka, Bangladesh; 6 Department of Disease Control, London School of Hygiene and Tropical Medicine, London, United Kingdom; Purdue University, UNITED STATES

## Abstract

**Introduction:**

Household drinking water can be contaminated by diarrheagenic enteropathogens at numerous points between the source and actual consumption. Interventions to prevent this contamination have focused on preventing exposure to human waste through interventions to improve drinking water, sanitation and hygiene (WASH). In many cases though, the infectious agent may be of zoonotic rather than human origin suggesting that unsafely managed animal waste may contribute to the contamination of household drinking water and the associated diarrheal disease burden.

**Methods:**

A cross-sectional household survey of 800 households was conducted across three informal peri-urban neighborhoods of Kisumu, Kenya, collecting stored drinking water samples, administering a household survey including water, sanitation and hygiene infrastructure and behaviors, and recording domestic animal presence and ownership. We used multivariate logistic regression to assess the association of traditional WASH factors and domestic animal presence and ownership on microbial contamination of household drinking water.

**Results:**

The majority of households sampled had fecally contaminated drinking water (67%), defined by the presence of any colony forming units of the fecal indicator bacteria enterococci. After adjustment for potential confounders, including socio-economic status and water and sanitation access, both household animal ownership (aOR 1.31; CI 1.00–1.73, p = 0.05) and the presence of animal waste in the household compound (aOR 1.38; CI 1.01, 1.89, p = 0.04) were found to be significantly associated with household drinking water contamination. None of the conventional WASH variables were found to be significantly associated with household drinking water contamination in the study population.

**Conclusions:**

Water, sanitation, and hygiene strategies to reduce diarrheal disease should consider the promotion of safe animal contact alongside more traditional interventions focusing on the management of human waste. Future research on fecal contamination of unsafe household drinking water should utilize host-specific markers to determine whether the source is human or animal to prepare targeted public health messages.

## Introduction

Safe water is required to sustain life, prepare food, and maintain personal and domestic hygiene [1]. However, a broad range of pathogenic micro-organisms, including viruses, parasites, bacteria, helminths, prions, and fungi, can be transmitted by water causing infection and disease [2,3,4]. Diarrheal disease, caused by a number of different enteropathogens, remains a leading cause of global child mortality and morbidity, especially in children under five and among the immunocompromised [5,6]. Approximately 34 million disability adjusted life years (DALYs) are lost each year due to unsafe water supplies in low- and middle-income countries (LMIC) [4]. Interventions to improve access to safe drinking water and sanitation, and interventions to improve hygiene behaviors such as hand washing with soap after defecation, have been shown to reduce the risk of diarrheal disease [1,7].

Many of these diarrhoegenic enteric infections however can be zoonotic and recent studies have highlighted the presence of animals in the domestic environment as a potential source of food and water contamination and a possible cause of diarrheal disease [8,9,10,11]. Animal husbandry when combined with poorly protected water supplies, a lack of water treatment at the source and/or at point of use, and poor housing conditions, particularly in areas of high population density where humans and animals cohabit, may be an important source of contamination and associated disease [9,10,12,13].

Although 2.6 billion people gained access to an improved drinking water source under the Millennium Development Goals (MDGs), almost a billion remain without access to this basic level of service [14,15]. Furthermore, access to an ‘improved’ drinking water source alone does not necessarily remove the risk of consuming contaminated water. One systematic reviewed reported that over a quarter of water sources classified as “improved” remained fecally contaminated [16]. These findings suggest that a large proportion of the world’s population remain exposed to contaminated drinking water, even when using an improved source [17,18,19]. The new Sustainable Development Goal (SDG) for water continues to call for “universal and equitable” access to safe water but also advocates for the improvement of drinking water quality [15].

Enterococci is a bacterial group found in human and animal waste and has been used as a common fecal indicator organism for the global characterization of safe recreational and drinking water according to the World Health Organization (WHO) [20,21,22]. The presence of enterococci demonstrates a recent fecal contamination from a warm-blooded animal or human host [20,22]. Enterococci persists longer in water and is more tolerant to chlorination and desiccation than *E*. *coli* [22]. Enterococci is the primary measure used for safe drinking water standards of the European Union (EU) [20,21,23].

The consumption of safe drinking water at the household level depends not just on the quality of water “at source” but also at the “point of use” and all stages between (i.e. transport and storage). At all points in this process there is a risk of contamination by both human and animal waste, and an associated risk of enteric pathogen exposure, infection and disease. The aim of this study was to assess whether household water contamination was associated with factors related to water, sanitation, hygiene (WASH), or animal presence in Kisumu, Kenya.

## Methods

**Fig 1:**
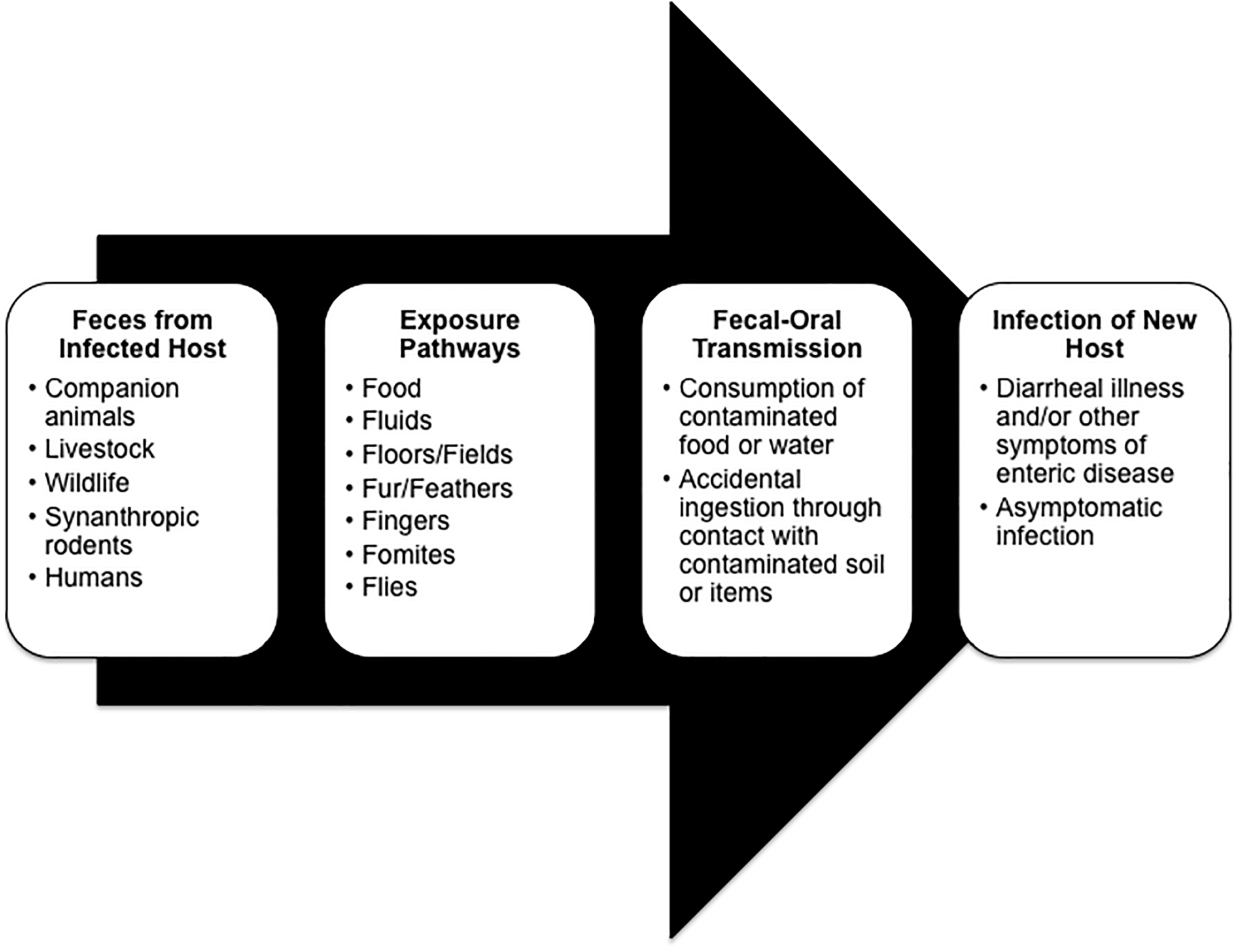
WASH and animal factors contributing to diarrheal illness. Adapted from Penakalapati G, Swarthout J, Delahoy MJ, McAliley L, Wodnik B, Levy K, et al. Exposure to Animal Feces and Human Health: A Systematic Review and Proposed Research Priorities [Internet]. ACS Publications; 2017, p. 11542.

A conceptual diagram of contributing factors that can lead to diarrheagenic enteropathogen exposure was developed to guide data collection and analysis (Fig 1). This diagram outlines both the traditional WASH components and animal contact as potential risk factors for diarrheal disease. In this study, water quality, sanitation, hygiene, and animal factors were examined for their relationship to household drinking water contamination.

### Study setting

This study was conducted in three informal peri-urban neighborhoods of Kisumu, Kenya: Nyalenda A, Nyalenda B and Kanyakwar. The Kanyakwar site included the areas of Nyawita and Obunga. Kisumu is the third largest city in Kenya with approximately 500,000 residents of whom 60% live in informal settlements or ‘slums’ [24]. Within these communities, a large proportion of housing is temporary or semi-permanent and occupied largely by tenants with limited access to safe water and sanitation [25,26]. Population growth within the informal settlements of Kisumu has not been met with the necessary increase in safe water and sanitation infrastructure [27]. It is estimated that half of peri-urban households in Kisumu are involved in some form of agriculture or animal husbandry [24,28].

Domestic animals commonly roam public spaces and residential compounds [26]. Livestock keepers predominately graze their animals in open, community spaces including government land, roadsides, private land plots, and rubbish sites [29]. Animal waste was disposed of through creating mounds of manure next to the animal(s) housing or moving it to public and open spaces away from the household [30]. Animal manure is often used in Kisumu for crop and garden fertilizer and contamination of household drinking water and food products has been theorized [30,31]. A recent study on domestic animal ownership in these peri-urban communities of Kisumu reported that whilst 34% of participating households had self-reported animal ownership, over 70% of the households had a domestic animal observed in the compound during the time of sampling [32]. Reasons given by households for animal ownership was most often for production of meat/eggs, as a source of income, or as pets [32]. Cohabitation with animals at night was commonly reported.

### Study design

During February and March of 2015, 800 randomly selected households were surveyed as part of a larger cross-sectional study investigating the impact of socioeconomic and household WASH disparities on enteric disease risk among children up to three years of age. The goal of the overall project was to examine the role of household characteristics, WASH equity, and environmental conditions on child health. The current study analyzed data related to household characteristics and WASH and domestic animal factors to determine associations with household drinking water contamination. Previous research among livestock keepers Kisumu found multiple risk factors for zoonotic disease exposure such as not treating drinking water prior to use, home slaughter of animals, and unsafe management of human and animal waste at the household [30].

Households were randomly selected using a two-stage cluster sampling design by which researchers received lists of current Community Health Volunteers (CHVs) representing the study communities and where each CHV is responsible for approximately 100 households. From these lists, 40 community clusters were firstly selected at random and then 20 households were selected at random from the CHV household list of each chosen cluster, using a random number generator in both cases. In all, 260 households were selected in Nyalenda A, 261 selected in Kanyakwar and 279 selected in Nyalenda B.

### Data collection

#### Household questionnaire and observations

Local field enumerators recruited by Great Lakes University Kisumu (GLUK) were trained to conduct the household and environmental surveys covering topics related to household and community water, sanitation, and hygiene characteristics and behaviors as well as domestic animal contact. Enumerators also collected observational data on household WASH characteristics. Following informed, written consent, the survey was delivered in English, Kiswahili, and Dholuo, depending on respondent’s preference, using handheld tablets and Qualtrics 2015 software. The survey instrument was written primarily in English but included translations for each question in the local Dholuo language [33]. As part of the inclusion criteria for the larger study, selected survey respondents were self-identified as the person in charge of water collection, hygiene, and infant food preparation for the household. If the respondent was not the head of the household, he/she answered questions to describe their characteristics. As in previous studies, a household was defined as those who share the same kitchen area [32,34].

#### Animal waste observations

The day after the survey was conducted, a second team of field staff revisited the households to record observational data on domestic animal and animal waste presence within the compound where the household is based. The second field team did not know the household survey results from the previous day. A transect walk between the two most distal points of the compound’s perimeter was undertaken and animal and animal waste presence were recorded. Observational data on animals and animal waste in the compound was linked to each surveyed household belonging to that compound.

#### Household water samples

In addition to the survey, participants provided a household drinking water sample to be analyzed for fecal contamination using the fecal indicator bacteria, Enterococci. The enumerator asked the respondent to pour no less than 500 mL of drinking water into a sterile Whirl-Pak® water sampling bag using the method they typically use to obtain water from the container (ex. respondent hands, cup, or ladle). Samples were labeled, placed in a cool box with ice to maintain a temperature 4–8°C, transported to the GLUK laboratory and processed within 6 hours after collection.

For enumeration of enterococci, 100 ml water samples were filtered through a 0.45μm pore size membrane filter (Millipore Corp., Bedford, MA, USA) and the filters were placed on a petri dish containing prepared media of Enterococcus Agar. This media is specific for the selection and enumeration of enterococci using membrane filtration following incubation at 35° C +/- 0.5° for 48 hours. Following this, all light and dark red colonies were counted as enterococci using a fluorescent lamp and magnifying lens and expressed as colony forming units (CFUs) present per 100 ml water samples. Positive and negative controls plates were used to verify results.

The logistic regression models used a binary variable to measure household drinking water contamination. Household drinking water contamination was created based on the results of the laboratory analysis on the presence of enterococci. Contamination was present (1) if there were colony forming units (CFUs) on the sample plate as recorded by the laboratory technicians. WHO guideline values state that any water for human consumption with > 0/100mL enterococci CFUs is contaminated or unsafe. This study elected to use the same cutoff values of 0 CFUs per 100mL to be safe and represent a zero risk for diarrheal disease while samples with ≥ 1 CFU enterococci per 100mL are contaminated and present a low to high risk for diarrheal illness [35]. In the event there were no recorded enterococci CFUs in the sample, the value was assigned a ‘0’ for absence.

### WASH and animal factors

Access to improved water and sanitation as well as protective hygiene behaviors have been associated with decreased water contamination and enteric disease exposure [1,7,13,36,37]. Using Prüss-Ustün et al.’s 2014 retrospective analysis of the impact of water, sanitation, and hygiene on disease in low- and middle-income countries as a guide, WASH conditions present in the household survey were examined with connection to household water contamination [4]. Categories of WASH variables included sanitation, hand hygiene, and water quality (Table 1) [1,38,39,40]. Improved drinking water sources included piped water into household or compound, public tap or standpipe, tube well or borehole while unimproved water encompassed cart with small tank or surface water [14].

**Table 1:** Variable summary of WASH and animal factors and covariates used to determine association with household drinking water contamination.

Sanitation	• Human waste disposal habits^a^ • Household access to improved sanitation^a^ • Child feces disposal habits^a^ • Open defecation sites nearby the home^a^
Hygiene	• Hand washing: 1) before eating; 2) After eating; 3) After toilet visit; 4) After picking up rubbish; 5) After handling dirty things; 6) After greeting people; 7) Before cooking; 8) Before preparing child’s food; 9) After changing baby; and 10) After handling animals • Presence of water basin with soap for hand washing^a^^,^^b^
Water Quality	• Household access to an improved water source^a^ • Reported drinking water treatment^a^ • Presence of lid on drinking water storage container^a^^,^^b^
Animal Factors	• Reported household animal ownership^a^ • Reported household rodent evidence in the past week^a^ • Recorded presence of domestic animals or animal waste in the compound^a^^,^^b^
Covariates	• Community where the household is located^c^ • Ownership or rental of residence^a^ • Wealth tercile for household calculated using a scale of household assets (*electricity*, *cooking fuel*, *household possessions*, *and access to a bank account*) and housing structure (*number of people per sleeping room and type of roof*, *wall*, *and floor*)^d^

^a^Recoded into binary variable indicating presence or absence for each household

^b^Reported through enumerator observation and not household survey

^c^Categorical variable indicating Nyalenda A, Nyalenda B, or Kanyakwar

^d^Categorical variable indicating poor, middle or rich.

Since animal waste can contribute to water contamination and disease transmission, analyses included determinants related to the presence of domestic animals in the compound and the household [41]. Variables were selected to represent potential exposure risks related to contact with domestic animals and rodent vectors. For the purposes of this research, animal contact was defined as: a) having direct interaction with an animal, animal waste, animal tissue or animal products; and b) sharing the same physical environment such as a home, yard/compound or community space [32]. In addition, the multivariate logistic regression model was adjusted for underlying determinants of household water contamination. A wealth asset variable was created similar to that used by the Kenya Demographic and Household Survey [34].

### Statistical analysis

Data collection and analysis were guided by the conceptual model (Fig 1) and data was analyzed using STATA® Statistical Software version 14.2 (Statacorp, 2017). Descriptive analysis was used to characterize households with and without water contamination as well as overall demographics, WASH conditions and behaviors, and domestic animal contact. The dataset was weighted to account for the number of clusters selected in each community and number of replaced or active CHVs within each cluster and again for the number of households listed on each CHV register and the number of households selected for participation.

Bivariate and multivariate logistic regression analysis tested for significant correlations between household drinking water contamination and variables related to water, sanitation, hygiene, and animals using significance levels of *p* value ≤ 0.05. An unadjusted bivariate regression model was used to determine statistically significant associations between factors classified as “WASH” and “animal” risk factors and household drinking water contamination, defined by the presence of any colony forming units of the fecal indicator bacteria enterococci. The multivariate model adjusted for household community, residence ownership, and wealth tercile. Data were assumed to be missing at random and analysis was conducted only on available data.

### Ethical approval

All subjects provided informed, written consent to participate in this study. Approval for this study was granted through the ethics committees of the London School of Hygiene and Tropical Medicine (LSHTM) [Ref No. 8482] and Great Lakes University Kisumu (GLUK) [Ref No. GREC/167/36/2014]. This study was exempt under the University of Florida’s Institutional Animal Care and Use Committee (IACUC).

## Results

### Household characteristics

In the majority of sampled households (67%), drinking water was fecally contaminated (Table 2). Proportions of contaminated water samples were greater in Kanyakwar (73%) and Nyalenda B (71%) than in Nyalenda A (57%). In households with water contamination, the person identified as the head of the household had most often finished primary school (69%) or secondary school (63%). Household heads were almost all employed (92%) and among those who were not employed, the majority had drinking water contamination in their home (73%). Household drinking water contamination was predominant across all wealth terciles.

**Table 2:** Characteristics of peri-urban households in Kisumu, Kenya with drinking water contamination (n = 505) and without (n = 291).

Category			Households with contamination	Households without contamination	Total
			n (%^+^)	n (%^+^)	n
Total			505 (67)	291 (33)	796***
Community					
	Kanyakwar		165 (19)	95 (23)	260
	Nyalenda A		147 (17)	114 (25)	261
	Nyalenda B		193 (64)	82 (52)	275
Level of education for head of household					
	Some primary		67 (13)	30 (10)	97
	Finished primary		228 (43)	120 (39)	348
	Finished secondary		157 (31)	105 (36)	262
	Post secondary		53 (13)	36 (15)	89
Level of education for respondent					
	Some primary		113 (24)	45 (14)	158
	Finished primary		264 (51)	164 (56)	428
	Finished secondary		93 (17)	55 (19)	148
	Post secondary		35 (8)	27 (12)	62
Gender of head of household*					
	Male		336 (71)	183 (64)	519
	Female		148 (29)	94 (36)	242
Gender of respondent					
	Male		42 (9)	22 (7)	64
	Female		463 (91)	269 (93)	732
Occupation of head of household					
	Not employed		45 (9)	19 (7)	64
	Employed		460 (91)	272 (93)	732
Occupation of respondent					
	Not employed		185 (37)	120 (43)	305
	Employed		320 (63)	171 (57)	491
Wealth tercile**					
	Poor		182 (33)	84 (28)	266
	Middle		167 (32)	97 (30)	264
	Rich		155 (36)	109 (42)	264
**Sanitation**					
	Have access to improved sanitation		36 (10)	27 (11)	63
	Have access to a toilet in compound		482 (97)	268 (94)	750
	Safe Disposal of Human Waste				
		Paid toilet in community	41 (8)	29 (10)	70
		Friend or neighbor’s house	348 (70)	204 (74)	552
		Public or school latrine	98 (21)	41 (16)	139
		Put child feces in latrine	356 (95)	202 (97)	558
		Bury child feces	56 (11)	25 (9)	81
		Place child feces outside of compound	18 (5)	8 (3)	26
		Child uses latrine	11 (4)	4 (2)	15
	Unsafe Disposal of Human Waste				
		Somewhere on compound grounds	18 (4)	8 (3)	26
		In a container at home	19 (5)	12 (4)	31
		In a bush/field	133 (31)	63 (24)	196
		In an open drain nearby	24 (5)	9 (5)	33
		In a nearby water source	7 (2)	6 (3)	13
		Put child feces in garbage in bag	50 (9)	27 (9)	77
		Put child feces in garbage without bag	23 (5)	14 (6)	37
		Leave child feces in yard/do nothing	30 (8)	13 (4)	43
		An open defecation site nearby	155 (32)	77 (26)	232
**Hygiene**					
	Observed hand washing basin with soap		228 (49)	135 (49)	363
	Household reported hand washing				
		Before eating	466 (92)	264 (91)	730
		After eating	358 (70)	196 (68)	554
		After toilet visit	462 (92)	263 (91)	725
		After picking up rubbish	178 (34)	107 (34)	285
		After handling dirty things	214 (41)	134 (45)	348
		After greeting people	124 (22)	84 (28)	208
		Before cooking	280 (55)	163 (54)	443
		Before preparing child’s food	296 (81)	177 (84)	473
		After changing baby	129 (23)	84 (31)	213
		After handling animals	8 (2)	4 (2)	12
**Water supply**					
	Have access to improved water		503 (99)	289 (99)	792
	Household drinking water source				
		Piped water to household	18 (5)	17 (8)	35
		Piped water to compound	110 (28)	45 (20)	155
		Public tap/standpipe	368 (65)	226 (71)	594
		Tube well/borehole	7 (1)	1 (<1)	8
		Cart with small tank	1 (<1)	2 (<1)	3
		Surface water	1 (<1)	0 (0)	1
**Water quality**					
	Reported treating drinking water		227 (52)	131 (51)	358
	An observed lid on water container		420 (82)	249 (84)	669
**Animal Factors**					
	Households with animal ownership		171 (37)	78 (29)	249
	Households that own poultry (duck or chicken)		110 (63)	51 (66)	161
	Households that own livestock (cattle, horse, pig, sheep, or goat)		81 (47)	36 (55)	117
	Households that own companion animal (cat or dog)		87 (51)	38 (47)	125
	Households with observed animal(s) in compound		366 (74)	218 (77)	584
	Households with observed animal waste in compound		368 (77)	195 (67)	563
	Household with rodent evidence during past week		326 (62)	182 (61)	508

*Reported head of household gender n = 761

**Reported households with wealth tercile n = 794

***Reported households with enterococci colony counts of drinking water n = 796

^**+**^Percentages based on weighted data.

Almost all households in the study reported access to improved water (n = 792) and over 90% of households have access to a toilet in the compound. Despite this, popular methods of unsafe human excreta disposal included somewhere on the compound grounds, in a container at home, and in open drains and water sources. Additionally, child excreta was commonly left in the yard, buried, or put into the garbage with or without a bag.

Almost half of the households had an observable basin with soap for washing hands (49%). Drinking water contamination rates were similar in households with improved hand washing facilities (37% of households had contamination and 33% did not). Reported events for hand washing were highest prior to eating, after visiting the toilet, and in households with a child (n = 589), before preparing child food. Only 2% (n = 12) of households reported washing their hands after handling animals.

Households reported owning cattle, horses, pigs, sheep, goats, chickens, ducks, dogs, and cats. Additional domestic animals observed in the household compounds include rabbits and pigeons. Domestic animal factors such as ownership, type of animal owned, presence of animal and/or animal waste, and evidence of rodents in the past week varied between households with and without contamination. Of households with reported animal ownership and data on drinking water contamination (n = 249), 37% of households with contamination also reported animal ownership compared to 29% of households without contamination. Observed domestic animal waste in the compound correlated to higher rates of water contamination but evidence of rodents in the home showed little difference between households.

Reported household human and animal drinking water sources were outlined in Table 3 according to community. The primary drinking water source for households across all communities was a public tap/standpipe (n = 597). In Nyalenda B, 33% of households had water piped to their housing compound (n = 90) and 10% of households had water piped directly to the home (n = 30). Twenty-two percent of households in Kanyakwar (n = 57) had water piped to their housing compound. Only four households used unimproved water sources such as cart with small tank (Kanyakwar n = 3) or surface water (Nyalenda B n = 1). In households that reported animal ownership, the primary source of drinking water for the animals was piped water (n = 129) followed by a stream/river (n = 79).

**Table 3:** Household drinking water source (n = 800) in three peri-urban communities of Kisumu, Kenya compared to the type of water source reported for household domestic animals (n = 252) within the same communities.

Category		Kanyakwar	Nyalenda A	Nyalenda B	Total
		n (%^a^)	n (%^a^)	n (%^a^)	n
**Household drinking water source**					
**Improved:**		**257 (20)**	**261 (20)**	**278 (60)**	**796**
	Piped water to household	5 (2)	0 (0)	30 (10)	35
	Piped water to compound	57 (22)	9 (3)	90 (33)	156
	Public tap/standpipe	194 (75)	247 (95)	156 (56)	597
	Tube well/borehole	1 (0.5)	5 (2)	2 (1)	8
**Unimproved:**		**3 (1)**	**0 (0)**	**1 (0.3)**	**4**
	Cart with small tank	3 (1)	0	0	3
	Surface water	0	0	1 (0.3)	1
**Domestic animal water source**^b^					
	Piped water	33 (44)	32 (50)	64 (58)	129
	Tube well/borehole	9 (12)	11 (17)	16 (16)	36
	Stream/river	29 (39)	22 (34)	28 (22)	79
	Other still surface water	3 (4)	0	4 (4.2)	7
	Drains	0	0	1 (0.5)	1

^a^Percentages based on weighted data

^b^More than one animal water source was indicated for households with reported ownership

### Multivariate analysis of correlates of water contamination

In bivariate analysis, no WASH factors were significantly associated with contamination of stored household drinking water (Table 4). Two animal related variables were significantly associated with contamination of stored household drinking water: households reporting animal ownership (OR 1.44; CI 1.07–1.96, p = 0.01); and, the observed presence of animal waste in the compound at the time of sampling (OR 1.60; CI 1.16–2.20, p = 0.02).

**Table 4:** Bivariate analysis of the association between WASH and animal factors and contamination of stored household drinking water.

Variable	Unadj. bivariate regression			Adj. multivariate regression		
	OR (95% CI)	Std. Err.	*P* value	aOR (95% CI)	Std. Err.	*P* value
**Sanitation**						
Have access to improved sanitation	0.86 (0.58–1.28)	0.17	0.46	0.79 (0.45–1.42)	0.23	0.43
An open defecation site nearby	0.75 (0.54–1.03)	0.12	0.08			
**Hygiene**						
Observed hand washing basin with soap	1.01(0.72–1.40)	0.16	0.96			
Household reported hand washing						
Before eating	1.07 (0.54–2.13)	0.36	0.83			
After eating	1.09 (0.76–1.54)	0.19	0.60			
After toilet visit	1.13 (0.50–2.53)	0.45	0.76			
After picking up rubbish	1.00 (0.72–1.39)	0.16	0.99			
After handling dirty things	0.83 (0.59–1.18)	0.14	0.30			
After greeting people	0.75 (0.54–1.04)	0.12	0.09			
Before cooking	1.06 (0.75–1.50)	0.18	0.74			
Before preparing child’s food	0.79 (0.50–1.25)	0.18	0.30			
After changing baby	0.67 (0.44–1.02)	0.14	0.06			
After handling animals	1.16 (0.26–5.13)	0.85	0.85			
**Water quality**						
Access to improved water source	1.31 (0.18–9.71)	1.29	0.79	1.53 (0.34–6.93)	1.14	0.57
Reported water treatment	1.03 (0.71–1.50)	0.19	0.88			
Water storage vessel has lid	0.85 (0.57–1.27)	0.17	0.42			
**Animal factors**						
Animal ownership	1.44 (1.07–1.96)	0.22	0.02*	1.31 (1.00–1.73)	0.18	0.05**
Animal presence	0.85 (0.52–1.37)	0.20	0.48			
Animal waste	1.60 (1.16–2.20)	0.25	0.01*	1.38 (1.01–1.89)	0.21	0.04**
Rodent evidence	0.96 (0.64–1.43)	0.19	0.84			
**Covariates**						
Household owns residence	1.45 (0.76–2.76)	0.46	0.25	1.31 (0.66–2.62)	0.45	0.43
Kanyakwar	-	-	**-**	-	-	-
Nyalenda A	0.79 (0.46–1.38)	0.22	0.41	0.77 (0.44–1.36)	0.22	0.36
Nyalenda B	1.47 (0.89–2.45)	0.37	0.13	1.46 (0.88–2.41)	0.36	0.14
Poor wealth tercile	-	-	-	-	-	-
Middle wealth tercile	0.91 (0.58–1.44)	0.21	0.69	1.03 (0.66–1.59)	0.22	0.90
High wealth tercile	0.75 (0.50–1.13)	0.15	0.16	0.74 (0.50–1.09)	0.14	0.12

*Significant at p ≤ 0.05 in the bivariate analysis

**Significant at p ≤ 0.05 in multivariate analysis

The two animal factor variables found to be significantly associated in the bivariate analysis were included in multivariate logistic regression models, adjusting for access to improved water and sanitation, community, household wealth tercile, and ownership of residence (Table 4). Both animal ownership (aOR 1.31; CI 1.00–1.73, p = 0.05) and observations of domestic animal waste in the household’s compound (aOR 1.38; CI 1.01, 1.89, p = 0.04) remained significant for household drinking water contamination after controlling for a number of potential confounding factors.

## Discussion

In these three peri-urban informal neighborhoods of Kisumu, Kenya, over 99% of households (796/800) had access to a water source classified as “improved” under the MDGs. Despite this, 67% of households (n = 505) had contaminated drinking water stored in the household at a WHO guideline value of >0/100mL enterococci CFUs. In bivariate analysis of a range of WASH and animal risk factors for contamination of stored drinking water, no WASH factors were found to be significantly associated but two animal factors–presence of animal waste in the compound of the household, and ownership of animals by the household–were significant.

Interactions with domestic animals are often beneficial as they provide humans with a source of food and clothing, transportation, draft power and income generation, affection and companionship, assistance in hunting and herding, protection from threats, cultural and religious identities, conservation and search-and-rescue efforts, and therapeutic and disability support [42]. However, research demonstrates that domestic animal contact can also lead to human diarrhea and other illness, the pollution of soil and food products, and water contamination [10,11,12,43,44].

Domestic animals in and around the living space of a household present an exposure risk to fecal material, which can harbor zoonotic pathogens. A recent review on animal waste exposure and human health outcomes in relation to water, sanitation, and hygiene found that animal feces exposure risks mirror the traditional “F diagram” for fecal-oral transmission pathways- contamination of water sources, soil, food, flies, hands, and fomites [45,46,47]. In fact, modeling studies have estimated that domestic animals are the predominate source of fecal pollution in water sources and, collectively, they create 85% of all global animal feces, significantly higher than that of humans [48]. Microbial source tracking of host-specific fecal contamination in Kenya has revealed cattle as the primary cause of watershed effluence [48,49]. Previous research on fecal contamination of water supplies in Kisumu found 95% of the samples had unsafe *E*. *coli* levels, which the authors believed was in part due to the presence of domestic animals in the informal settlements [26]. This hypothesis is supported by a recent study in rural Bangladesh housing compounds which found the proportion of fecal contamination in stored drinking water, on the hands of children under five, and in the soil where children under five play to be predominantly ruminant or avian as opposed to human [50].

Animal waste in community water sources exposes residents to enteric diseases through the ingestion of contaminated water and food products and through contact with recreational water [9,51]. In the home, water contamination can occur when drinking water is stored in containers that are not covered and are exposed to contact with humans, animals, and vectors [10,52,53]. Stored drinking water can also become contaminated post-treatment when pathogens are introduced from household members’ hands as they decant water to, or from, the storage vessel to secondary containers [10,54]. Thus point-of-use treatment can be as important in ensuring the microbiological quality of water as actions taken at the water source [7,36,38,55,56].

Poor water quality can influence the safety of food preparation, hand washing, and personal and household hygiene [39,52,57]. Hand washing is critical for the prevention of food and water contamination and has been estimated to prevent 47% of diarrheal disease risk [39]. Hand washing can also protect against exposure to enteric zoonoses found in animal waste [41]. Recent work in rural western Kenya found that children under five who washed their hands following animal contact had lower rates of moderate to severe diarrhea [11]. Additional personal behaviors such as proper food handling, safe disposal of human and animal waste, and personal cleanliness can also impact water quality and the risk of enteric diseases [13,58]. This study population demonstrated positive hygiene behaviors related to hand washing prior to eating, after using the toilet, and before preparing a child’s food. However, low performance was reported after picking up rubbish and after changing a baby. Almost no survey respondents reported washing hands after animal contact.

In less-developed areas, human exposure to animal waste is more widespread as domestic animals and their waste are not removed from living spaces [46]. In this study, the majority of households had observed animal waste in their living compound at the time of sampling although less than a third of the households reported domestic animal ownership. This demonstrates a lack of animal waste management and movement restriction, which can lead to individual and community-level exposures to animal feces and potential diarrheal illness.

Traditional WASH-related studies and interventions have focused on water quality, access to proper sanitation, and the promotion of protective hygiene behaviors as a triad for the prevention of enteric diseases [1,7,59]. However, this study showed a significant association between household drinking water contamination and domestic animal factors but found no relationship to the commonly measured WASH characteristics. Households with animal ownership were significantly associated with water contamination as was the observed presence of animal waste in the compound. This research establishes the need for more discussion on the merits of including an even larger set of WASH intervention messages related to safe contact with animals in the home and the community. More research is needed on the impact of proper animal waste management practices on future projects aimed at reducing enteric diseases from unsafe drinking water.

### Study limitations

While the data analysis reveals a potential connection between water contamination and domestic animals, the relationship could not be assumed to be causal due to the cross-sectional data. False positives or variable significance in the model may also be due to chance as a result of multiple testing. Additionally, the WHO guideline of 0/100mL enterococci CFUs can be hard to attain in developing countries and bacterial exposure may not be indicative of true diarrheal disease risk [18,35]. Singular use of the fecal indicator organism enterococci instead of a host-specific pathogen could not delineate a zoonotic source for the water pollution.

Furthermore, due to the nature of the single transect walk, animal presence and waste in the compound could have been missed. The presence of domestic animals in these compounds may also have been underestimated as not all households in the compound were selected to participate in the survey. Future studies on the existence of peri-urban domestic animals should use observations at different times or for longer periods. Moreover, household animal ownership should be approached with a culturally-relevant definition to ensure that animal husbandry at the household level is not overlooked. Some household members may have decision-making abilities over the sale or food production of an animal but would not consider themselves the animal’s owner. The authors recommend focus group discussions with community members to determine how best to tackle issues of animal ownership and husbandry in these peri-urban communities.

## Conclusion

Urban and peri-urban animal husbandry is on the rise to meet increasing demands for milk and meat products, which can provide much-needed protein and energy rich foods and serve as additional income for poorer households, including those of Kisumu [11,60–62]. However, with intensified contact with animals and animal products, people are increasingly exposed to zoonotic disease risks at the household and community level. Our study underscores the importance of considering animal sources of drinking water contamination and as a potential disease risk. New strategies may consider including messages related to the risk of zoonotic diseases as well as the importance of using improved water sources, maintaining adequate point-of-use treatments, utilizing quality sanitation facilities and removing human and animal waste from living spaces, and the promotion of personal hygiene measures such as hand washing. In informal peri-urban and urban areas with high population density combined with a high prevalence of animal ownership these risks are likely greater and the need for effective strategies more acute.
